# Diurnal Emotional States Impact the Sleep Course

**DOI:** 10.1371/journal.pone.0142721

**Published:** 2015-11-25

**Authors:** Julien Delannoy, Osamu Mandai, Jacques Honoré, Toshinori Kobayashi, Henrique Sequeira

**Affiliations:** 1 SCALab, CNRS UMR 9193, Université de Lille, Lille, France; 2 Sleep Research Center, Ashikaga Institute of Technology, Ashikaga, Japan; 3 Neurosciences, UFR Biologie, Université de Lille, Lille, France; Oasi Institute for Research and Prevention of Mental Retardation, ITALY

## Abstract

**Background:**

Diurnal emotional experiences seem to affect several characteristics of sleep architecture. However, this influence remains unclear, especially for positive emotions. In addition, electrodermal activity (EDA), a sympathetic robust indicator of emotional arousal, differs depending on the sleep stage. The present research has a double aim: to identify the specific effects of pre-sleep emotional states on the architecture of the subsequent sleep period; to relate such states to the sympathetic activation during the same sleep period.

**Methods:**

Twelve healthy volunteers (20.1 ± 1.0 yo.) participated in the experiment and each one slept 9 nights at the laboratory, divided into 3 sessions, one per week. Each session was organized over three nights. A reference night, allowing baseline pre-sleep and sleep recordings, preceded an experimental night before which participants watched a negative, neutral, or positive movie. The third and last night was devoted to analyzing the potential recovery or persistence of emotional effects induced before the experimental night. Standard polysomnography and EDA were recorded during all the nights.

**Results:**

Firstly, we found that experimental pre-sleep emotional induction increased the Rapid Eye Movement (REM) sleep rate following both negative and positive movies. While this increase was spread over the whole night for positive induction, it was limited to the second half of the sleep period for negative induction. Secondly, the valence of the pre-sleep movie also impacted the sympathetic activation during Non-REM stage 3 sleep, which increased after negative induction and decreased after positive induction.

**Conclusion:**

Pre-sleep controlled emotional states impacted the subsequent REM sleep rate and modulated the sympathetic activity during the sleep period. The outcomes of this study offer interesting perspectives related to the effect of diurnal emotional influences on sleep regulation and open new avenues for potential practices designed to alleviate sleep disturbances.

## Introduction

Diurnal emotional states are associated with night sleep quality in the daily life of most people. For example, people experiencing diurnal negative feelings, such as anxiety, irritability, or stress, often report poor sleep quality [1–6], insomnia [7], shorter sleep duration [8], more frequent waking [6, 9], as well as more Rapid Eye Movement (REM) sleep, shorter REM sleep latency and fewer Non-REM stages 3 and 4 (NREM3) [10]. In contrast, individuals experiencing feelings of well-being usually report better sleep quality [3, 11–14]. However, how pre-sleep emotional states modulate the structure of sleep has received far less attention (see Kim and Dimsdale [15] and Deliens *et al*. [16] for reviews). In addition, the available studies often have methodological limitations and most of them neglect pre-sleep positive emotional states. Moreover, these studies have rarely considered the nature and intensity of the stressful events, or the delay between these events and the sleep period. In fact, psychological or physical stressors could have different impacts on sleep parameters [17].

In order to alleviate these limitations, several authors have applied procedures designed to control the nature of the stressful event and its occurrence before sleep. For example, in order to induce a negative state at bedtime, one method used feelings of apprehension about the day following the sleep period. Such feelings were induced by telling the participant that he would be evaluated the next day [18], or by worrying him about some hard work [19]. In these conditions, consequent changes were observed, including a longer REM sleep latency, less REM during the last part of the night, a reduction in the percentage of NREM3 and an increase in Non-REM stage 2 (NREM2) sleep [18, 19]. Another procedure consisted in exposing participants to a near-impossible task in order to make them feel bad about their own intellectual capacities. Depending on the study, REM sleep proved to be affected by this kind of procedure. Thus, its percentage during the night decreased and it was interspersed with more awakenings [20], or REM sleep episodes were more frequent [21], or an increase in NREM2 and REM sleep and a decrease in NREM3 sleep at the beginning of the sleep period were reported [22]. Though these procedures seemed effective at inducing negative emotional states, the induction remained dependent on the experimenter’s behavior [23].

As an alternative, some authors have chosen to induce emotional states with emotional movies, which keep the participant blind to the aim of the procedure, reduce the involvement of the experimenter, and enable good control of the timing and efficiency of the induction [24]. Watching a negative movie just prior to the sleep period leads to more NREM2 sleep [25], a reduction in the percentage of REM sleep and more awakenings [26, 27]. It can also increase REM density [26, 27] or change the dream content during the earlier part of the night [28, 29]. Concerning positive movies, the scarce data suggest that positive emotional states prior to sleep can improve sleep problems [30], but the details of this influence remain unexplored.

In order to investigate sleep architecture further following emotional events, autonomic nervous system (ANS) indicators could be of valuable interest. In particular, the sympathetic division of the ANS is strongly associated with central activations required by the processing of cognitive and emotional information (see Kreibig [31] for a review). Among autonomic indicators, electrodermal activity (EDA), related to the activity of eccrine sweat glands exclusively under sympathetic control, has proved to vary very specifically through sleep stages in humans [32–35] and cats [36, 37], suggesting different levels of sympathetic activation during these stages. The frequency of electrodermal responses (EDRf) is high during NREM3 sleep, very low during REM sleep, and has medium values in NREM2 and NREM1 stages. EDA is known to be particularly relevant to the study of central arousal linked to attention, novelty and especially emotion. Thus, it has been widely used as a robust marker of emotional processes in awake states (see Critchley [38], and Sequeira *et al*. [39] for reviews). Moreover, EDA has been associated with cognitive processes during sleep [40]. Surprisingly, however, there is only very sporadic research on sleep attempting to consider electrodermal variations as potential indicators of sleep processes related to pre-sleep emotional states. In a few studies, a higher EDR frequency has been proposed as a marker of pre-sleep anxiety or stress states, during NREM3 sleep [34, 41, 42] or of the bizarreness of dreams during REM sleep [43].

Taken together, these data strongly suggest that diurnal emotions can induce changes during the following sleep period, especially during REM and NREM3 sleep stages [15]. Although there is evidence that a pre-sleep negative emotional state affects sleep parameters, there is no consensus about the pattern of such effects. This could be due to the variety of the inducing events or stimulations. Furthermore, there are no consistent data about the impact of pre-sleep positive emotional states on the sleep course. Finally, recording sympathetic indicators of emotional processing during the sleep period could improve the characterization of the impact of diurnal emotions on sleep organization.

Accordingly, the present research has a double aim: firstly, to identify the specific effects of pre-sleep emotional states on REM and NREM3 stages of the subsequent sleep period; secondly, to relate such states to the sympathetic activation during the same sleep period. In accordance with the literature, we postulate that pre-sleep emotional states will significantly impact the sleep architecture and sympathetic activation. In order to test these hypotheses, we developed and validated emotional and non-emotional movies as controlled pre-sleep emotional stimulations. Then, their effectiveness at eliciting pre-sleep emotional states was evaluated, before a normal period of sleep during which a polysomnogram and the EDA were recorded.

## Materials and Method

### Participants

Twelve healthy right-handed adults, all males and volunteers (age: 19–22 years; mean ± SD: 20.1 ± 1.0), participated in the experiment. They were recruited by advertising mail sent to a student mailing list of Ashikaga Institute of Technology (Tochigi, Japan). This study was approved by the local human ethics studies committee of Ashikaga Institute of Technology and each participant provided his written informed consent before being included.

There was one full week between the participant’s agreement and their first night in the laboratory. During this week, and for the full experimental period, participants were asked to maintain a normal sleep schedule (bedtime between 23:00 and 01:00, and waking up between 07:00 and 09:00), and to avoid napping. Compliance with these rules was monitored with a sleep diary and an actiwatch. They were also asked to avoid psychoactive substances (like caffeine). Participants did not complain about any physical or psychological health problems, as verified, during the first night, by two questionnaires: the Japanese version of the Cornell Medical Index (CMI) and the YG Personality Inventory (YGPI). Furthermore, participants did not report any current or previous sleep or psychiatric disorder, and did not take any medication from one week before the experimentation until its end.

### Material

#### Experimental environment

The sleep experimentation unit has two adjoining rooms. The experimenter room contained the recording equipment, which enabled the experimental procedure to be monitored. An infrared video camera, a microphone and an intercom were used to maintain visual and vocal contact with the participant who was installed in the other room. This was isolated, quiet and shielded, containing a bed, a projection screen (2.0 m wide x 1.4 m high), and a chair placed 2.0 m in front of the screen, where the participant was comfortably seated during the presentation of emotional stimulations.

#### Stimuli

Emotional and non-emotional movies were used to induce emotional states before sleep. To this end, a specific procedure was used to select video clips with the aim of obtaining three final validated movies corresponding to negative, neutral and positive valence, respectively.

In the first step, different videos were extracted from cinematographic movies, and a database of 63 pre-selected video clips was established (29 negative, 14 neutral, and 20 positive). The duration of each video clip varied between 1 and 4 minutes.

In the second step, the emotional impact of each of the 63 pre-selected extracts (mean duration: 2.0 ± 0.7 minutes) was evaluated by Japanese students, with a methodology similar to that used by Schaefer *et al*. [44]. Their valence and activation values [45] were rated, and the Japanese version of the *Positive And Negative Affect Scale* (PANAS) [46] was completed. In fact, three sets of 21 video clips were created, each comprising 9 or 10 negative, 4 or 5 neutral and 6 or 7 positive video clips. The order of the video clips in each set was randomized, with the condition that a clip never had the same valence as the preceding one. The sum duration per set was 42.1 ± 1.3 min. Each set was evaluated by a group of 11 to 13 students, who were not involved in the main sleep experiment. For each video clip, participants were asked to imagine themselves in the scene, as if it was real. Then, the participant evaluated his emotional state using two Self-Assessment Manikin Scales (SAM), developed by Bradley and Lang [47] to score the two main dimensions of emotion; valence (from 1, negative, to 9, positive) and arousal (from 1, low, to 9, high). The evaluation was completed by the PANAS, from which two subscores could be obtained: Positive Affect (PA) and Negative Affect (NA). Each clip was followed by a 5-minute break in order to complete the questionnaires and limit the persistence of the effect of the previous clip. A 15-minute break was provided every 10 video clips (*i*.*e*. every hour) for relaxation, during which participants were free to do as they wished.

In the third step, the video clips with the highest scores in their respective categories (negative, neutral and positive) were compiled with specific software *(Premiere Pro v5*.*5*, *Adobe)* to create three movies of corresponding valence. Each movie lasted 20.4 ± 0.2 minutes, started with the least arousing clip and ended with the most arousing one. A non-lyrical soundtrack was synchronized with the movie, matching the action and the emotion to reinforce the immersion [24].

The neutral movie was characterized by a mean valence of 5.2 ± 0.3, and an arousal of 2.0 ± 0.3. In comparison, the values of valence and arousal of the positive movie were higher (valence: 6.8 ± 0.5; t_17_ = 8.1; p < 0.001; arousal: 3.7 ± 0.5; t_17_ = 8.9; p < 0.001) whereas for the negative movie, the valence was lower (3.4 ± 0.8; t_17_ = 6.3) and the arousal higher (4.8 ± 1.1; t_17_ = 7.1; p < 0.001; Table 1).

**Table 1:** Scores of SAM and PANAS subscales for each movie. These scores (mean ± SD) were calculated from those obtained for different video clips constituting each movie. **n** is the number of video clips for each movie.

		Movie		
		Negative (n = 11)	Neutral (n = 8)	Positive (n = 11)
**SAM**	Valence	3.4 ± 0.8	5.2 ± 0.3	6.8 ± 0.5
	Arousal	4.8 ± 1.1	2.0 ± 0.3	3.7 ± 0.5
**PANAS**	Positive Affect	17.1 ± 2.4	16.5 ± 2.1	24.2 ± 2.1
	Negative Affect	25.6 ± 2.4	15.2 ± 2.5	14.8 ± 0.7

According to the PANAS, the neutral movie was characterized by a mean score of 16.5 ± 2.1 on the positive affect subscale and 15.2 ± 2.5 on the negative subscale. Compared to the neutral scores, the positive movie had a higher score on the positive affect subscale (PA = 24.2 ± 2.1; t_17_ = 7.68, p < 0.001), with no difference on the negative affect subscale (NA = 14.8 ± 0.7; t_17_ = 0.60, p = 0.55). The negative movie had a higher score on the negative affect subscale (NA = 25.6 ± 2.4; t_17_ = 9.34, p <0.001), with no difference on the positive affect subscale (PA = 17.1 ± 2.4; t_17_ = 0.44, p = 0.66). Positive and negative movies were also significantly different according to both subscales (NA: t_20_ = 7.33, p < 0.001; PA: t_20_ = 14.74, p < 0.001) but their respective scores at congruent subscales remained indistinguishable (NA–PA: t_20_ = 1.59, p = 0.13).

#### Recordings

Polysomnographic recordings included an electroencephalogram (EEG), an electrooculogram (EOG) and an electromyogram (EMG). The EEG activity was recorded with 6 tin electrodes (F3, F4, C3, C4, O1, O2), positioned according to the 10–20 system [48], using tin electrodes referenced to linked earlobes (impedance <1 kΩ). Four additional electrodes were placed at the outer canthi of each eye and above and below the right eye for a bipolar recording of the EOG, while two bipolar referenced electrodes for the EMG were placed on the chin. All electrodes were connected to a headbox (San Ei Electrode Box) linked to a recording system (San Ei Biotop 6512) enabling the filtering (0.5 Hz-100 Hz pass band), sampling (256 Hz) and amplification of signals, continuously processed during emotional induction and the sleep period using the software Sleep Maister 1.2 (IAC, Tokyo, Japan).

Electrodermal activity was recorded using the conductance technique (constant-voltage method, 0.5 V) at a sampling rate of 256 Hz, and low-pass filtered at 50 Hz. Two Ag-AgCl electrodes (active area: 1 cm in diameter) were attached to the palmar side of the middle phalanges of the second and third fingers of both hands. Electrodes were connected to a common acquisition system (*PowerLab and LabChart 7*, *AD instruments*) for amplification. A back-up of all signals was made with a link to a magnetic band recorder *(Cassette Data Recorder XR-7000L*, *TEAC)*.

### Procedure

Each participant slept for 10 nights undergoing polysomnographic recording. The length of each night never exceeded 10 h between electrode placement and withdrawal. On arrival, the participant completed the two questionnaires, *CMI* and *YGPI*. The first night helped the participants to become familiarized with the recording apparatus and the experimental room, and was intended to avoid the so-called first-night effect [49, 50].

**Fig 1:**
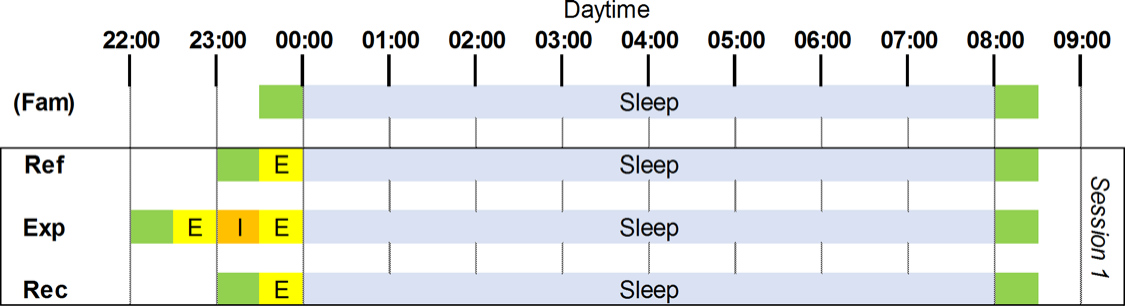
Schematic representation of the experimental protocol for the first four nights. Fam, familiarization night, exclusively before the first session; Ref, Reference night; Exp, Experimental night, during which one of the three movies was presented to the participant; Rec, Recovery night. In green, time to install or remove electrodes; in orange, emotional induction (I); in yellow, evaluation (E) of emotional state with PANAS; in blue, sleep period.

For each subject, the other 9 nights were grouped into 3 sessions, taking place in three successive weeks. Each one was constituted of 3 consecutive nights. The first night (reference: Ref) provided baseline recordings. During the second night, devoted to the experiment (Exp), one particular emotional state was induced. The third night (recovery: Rec) was added to observe the possible persistence of the induced emotional state (Fig 1).

During the Ref night, the participant arrived at 23:00. Electrodes were attached, and then the participant completed the PANAS. During the Exp night, the participant arrived at 22:00. The electrodes were attached, then the participant completed the PANAS a first time; at 23:00, to induce a particular emotional state before sleep, the participant watched one of the three movies. The order of the movies was counterbalanced across participants. After the movie, the emotional state was measured a second time using the PANAS, just before the sleep period. The organization of the Rec night was similar to that of the Ref night. Every night, at midnight, the participant was installed in bed and the lights were turned off. He was woken up after the last period of REM sleep, between the 7^th^ and 8^th^ hour after the lights were switched off.

During the week between two sessions and during the day between each night of one session, the participant was free to carry out his usual activities in compliance with the rules previously indicated.

### Data analysis

#### Psychometric questionnaire

The two PANAS subscores were extracted and transformed into z scores ([Value of raw score–Mean value of participant] / [Standard deviation of the participant]) for each participant and each measure.

#### Hypnogram

Sleep recordings were scored manually, by two experimenters, according to the standard rules of the American Academy of Sleep Medicine [51]. Based on epochs of 20 seconds [51], the following sleep variables were extracted: total sleep time (TST), sleep onset latency (SOL: from the moment the lights were turned off until the first minute of NREM2), waking after sleep onset (WASO), sleep efficiency (SE); percentage of stage 2 sleep (NREM2), stage 3 sleep (NREM3), and REM sleep after sleep onset; latency of NREM3 and REM sleep. Due to the absence of NREM1 after sleep onset for some nights, this sleep stage was not considered for analysis.

These variables were first extracted for the full night (*i*.*e*. between sleep onset and the last full cycle). Then, in order to highlight the possible dynamics of sleep variations, variables were also extracted according to the first and second halves of the night’s sleep. Epochs containing movements or EMG artefacts were excluded from the analysis.

#### Electrodermal activity

The full raw signal was first low-pass filtered (cutoff frequency 0.5 Hz, 32^nd^ order FIR filter). EDRs were extracted using validated homemade automated detection software (EDAnalysis). The amplitude of each response was calculated from the difference between the peaks and the starting conductance values. Every EDR with an amplitude higher than 0.02 μS was selected for further analysis. EDRf was calculated for each epoch without movements or EMG artefacts.

#### Statistical analysis

Two preliminary analyses were carried out, in which no differences were expected: 1) for each measure, the Ref nights preceding each kind of movie were compared to monitor the homogeneity between conditions; 2) for PANAS scores, at arrival on the Exp night, to control the absence of diurnal variation, which can play a role in the efficiency of the induction.

Concerning the PANAS, in addition to the analysis of subscale scores, the difference between both subscales (PA-NA) was also considered. It was positive when the PA score was higher than the NA score, and negative when it was lower.

The effect of emotion content was analyzed according to Lang’s model of emotion, based on arousal and valence dimensions [45]. The *Arousal effect* was modeled with a quadratic contrast (QC), opposing negative (+1) and positive (+1) with neutral conditions (-2). The *Valence effect* was modeled with a linear contrast (LC), opposing negative (-1) to positive (+1) and excluding neutral conditions (0). Planned comparisons were designed to assess the *induction* (Exp night—Ref night), the *persistence* (Rec night–Ref night) and the *recovery* (Rec night–Exp night) effects of the movies. Tests for the normality of the distributions, as well as ANOVAs and planned comparisons, were carried out using Statistica *(Statsoft)* software. The significance threshold was set at p = 0.05.

## Results

### Pre-sleep effects of emotional induction

Between Ref nights, no significant variation was found for NA (F_2,22_ = 2.07, p = 0.15) and PA (F_2,22_ = 0.09, p = 0.92) subscales, nor for the difference between PANAS subscales (F_2,22_ = 0.80, p = 0.46). Between arrivals on Exp nights, no variation was found for NA (F_2,22_ = 0.97, p = 0.40) and PA (F_2,22_ = 1.81, p = 0.19) subscales nor for the difference between them (F_2,22_ = 0.46, p = 0.63).

**Fig 2:**
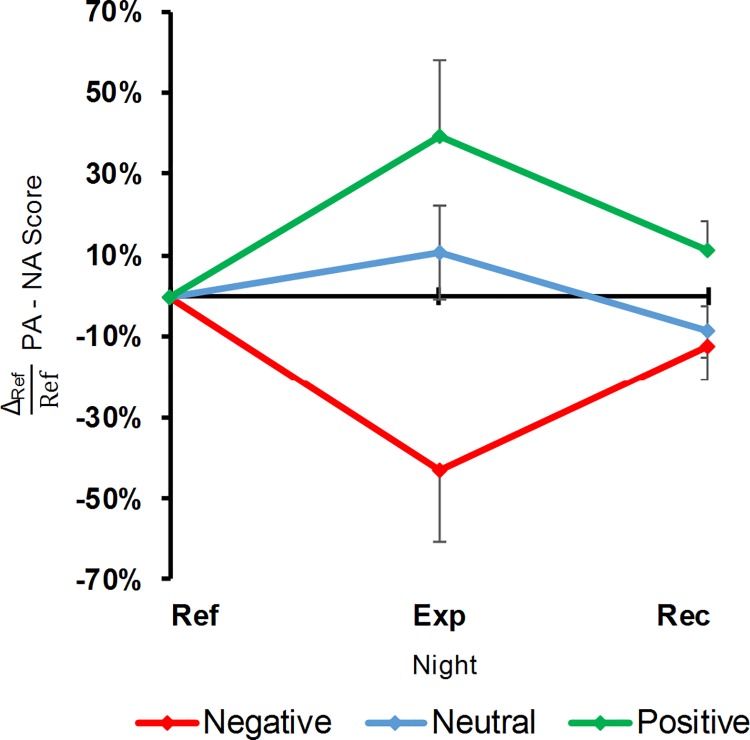
PANAS PA-NA scores as a function of movies and nights. Percentages of variation relative to the Ref night (Δ_Ref_; means and standard errors of means). Ref, Exp and Rec: Reference, Experimental and Recovery nights. Negative, Neutral and Positive: valences of the movies used for emotional induction. Note the valence effect observed for the Exp night and recovering for the Rec night (red and green curves).

Emotional induction prior to sleep resulted in a valence effect on the NA score (LC: F_1,11_ = 7.33, p = 0.02; QC: F_1,11_ = 2.97, p = 0.11), with higher scores after the negative movie than after the positive one. It also had a valence effect on the PA score (LC: F_1,11_ = 5.48, p = 0.04; QC: F_1,11_ = 0.65, p = 0.44), but this time the scores were higher after the positive movie. The persistence of these congruent effects was not significant (LC: F_1,11_ = 1.91, p = 0.19), whatever the subscale (LC: F_1,11_ = 0.03, p = 0.86), but the recovery was (LC: F_1,11_ = 6.43, p = 0.03), with no difference between the subscales (LC: F_1,11_ = 0.73, p = 0.41). All these effects were reflected by the PA-NA score (Fig 2), which was positive after the positive movie and negative after the negative one.

### Effects of pre-sleep emotional states on sleep architecture

Whether based on full or half-nights, the analyses did not reveal any significant change concerning SOL (all Fs_1,11_ < 0.21, ps > 0.65), TST (all Fs_1,11_ < 0.98, ps > 0.34), WASO (all Fs_1,11_ < 3.36, ps > 0.09), sleep efficiency (all Fs_1,11_ < 3.79, ps > 0.07), NREM3 latency (all Fs_1,11_ < 2.60, ps > 0.14), or REM sleep latency (all Fs_1,11_ < 2.53, ps > 0.14). Only sleep stage distribution varied as a function of the night and emotional induction.

The preliminary analyses of Ref nights did not reveal any significant variation in overall stage rates between experimental conditions: NREM2 (54.4 ± 9.0%, F_2,22_ = 0.18, p = 0.84), NREM3 (20.3 ± 6.0%, F_2,22_ = 0.30, p = 0.74) and REM (20.6 ± 4.9%, F_2,22_ = 2.25, p = 0.13). Moreover, the comparison of the two halves of Ref nights confirmed some established points. In the first part of the nights, there was less NREM2 (51.6 ± 11.2%), more NREM3 (31.1 ± 10.5%), and less REM sleep (12.5 ± 6.0%) than in the second part (NREM2: 57.3 ± 10.5%, F_1,11_ = 4.33, p = 0.06; NREM3: 9.5 ± 5.3%, F_1,11_ = 62.31, p < 0.01; REM: 28.7 ± 7.9%, F_1,11_ = 62.31, p < 0.01).

#### Full-night effects

**Fig 3:**
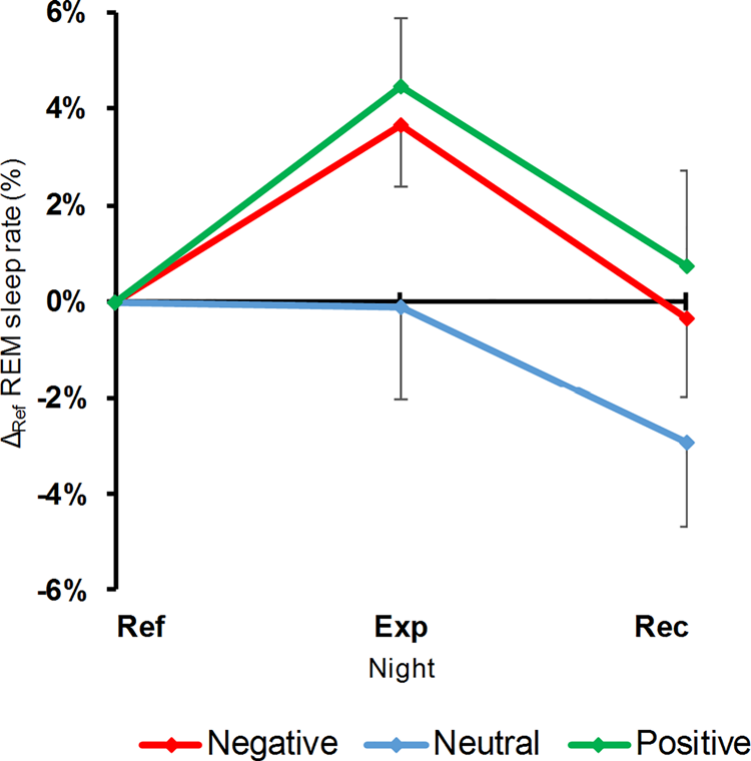
REM sleep rate variations, according to nights and movies. Differences (means and standard errors of means) between each night and its corresponding Ref night. Abbreviations for nights and movie valences are the same as those of Fig 2.

The rate of NREM3 was not modified by the induction procedure (LC: F_1,11_ = 0.11, p = 0.75; QC: F_1,11_ = 0.06, p = 0.82). On the contrary, the REM sleep rate showed an arousal effect of emotional induction (LC: F_1,11_ = 0.41, p = 0.54; QC: F_1,11_ = 5.35, p = 0.04): both emotional movies increased the REM sleep rate while the neutral one had no effect. Though this arousal effect was no longer significant for the Rec night (QC: F_1,11_ = 2.29, p = 0.16), this did not correspond to a significant recovery (QC: F_1,11_ = 0.52, p = 0.49; Fig 3).

When considering the two halves of the nights, there was no change in the emotional induction effect on the NREM3 rate (F_2,22_ = 0.82, p = 0.45). However, such a change did occur for REM sleep (F_2,22_ = 3.71, p = 0.04) and this dynamic is analyzed further in the following section.

#### Half-night effects on REM sleep

**Fig 4:**
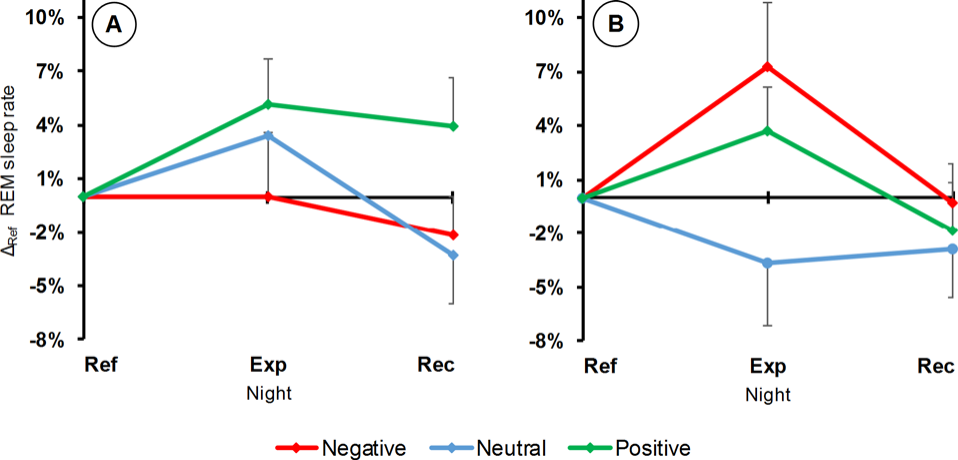
REM sleep rate variations according to half-nights, nights and movies. Differences between each half-night and its corresponding half of the Ref night (means and SEM). A: first half of the night; B: second half. Abbreviations for nights and movie valences are the same as those of Fig 2.

During the first half of the night (Fig 4A), the REM sleep rate tended to vary according to a valence effect (LC: F_1,11_ = 3.85, p = 0.07; QC: F_1,11_ = 0.12, p = 0.73), with an increase for the positive movie only. No persistence (LC: F_1,11_ = 2.76, p = 0.12) or recovery effects (LC: F_1,11_ = 0.01, p = 0.93) were found.

During the second half of the night (Fig 4B), the REM sleep rate showed a significant effect of emotional arousal after the induction procedure (LC: F_1,11_ = 1.51, p = 0.24; QC: F_1,11_ = 6.50, p = 0.03), with a higher rate following the emotional movies than following the neutral one. This effect did not persist (QC: F_1,11_ = 0.39, p = 0.55) till the Rec night and significantly recovered (QC: F_1,11_ = 13.11, p = 0.004).

### Effects of pre-sleep emotional states on sympathetic activity

**Fig 5:**
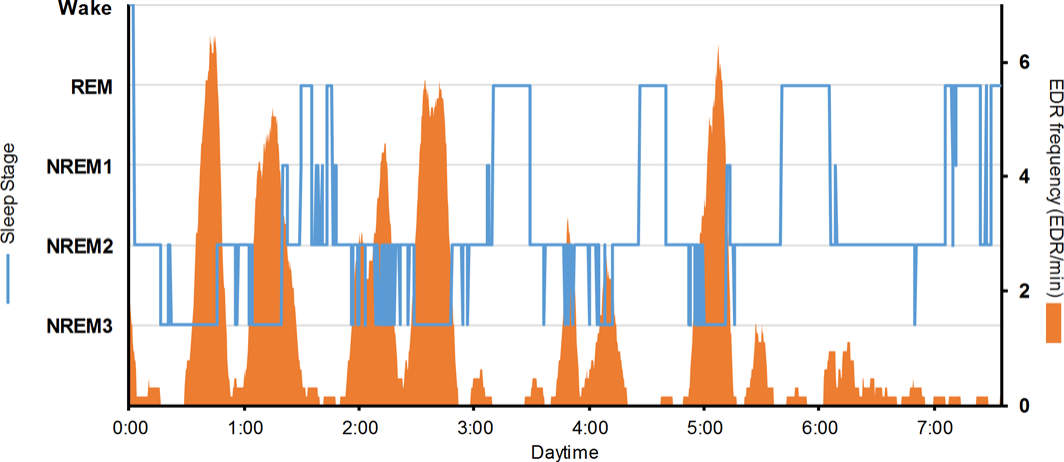
Typical example *(participant #2)* of the temporal distribution of sleep stages (blue) and EDRf (orange). Note that the EDR is clearly more frequent during the NREM3 stage.

The distribution of EDRs as a function of the sleep stage was compatible with the classic data (Fig 5). Across all nights and emotional conditions, the frequency of EDR was higher during NREM3 (1.0 ± 1.2 EDR/min) compared to NREM2 (0.5 ± 0.6 EDR/min; F_1,11_ = 9.08, p = 0.01) or REM (0.2 ± 0.2 EDR/min; F_1,11_ = 7.76, p = 0.02). The frequency also tended to be higher during NREM2 than during REM (F_1,11_ = 4.02, p = 0.07).

The preliminary analyses of Ref nights did not reveal any significant difference in EDRf, whatever the sleep stage, NREM2 (F_2,22_ = 0.50, p = 0.61), NREM3 (F_2,22_ = 0.60, p = 0.56) or REM (F_2,22_ = 0.64, p = 0.54).

**Fig 6:**
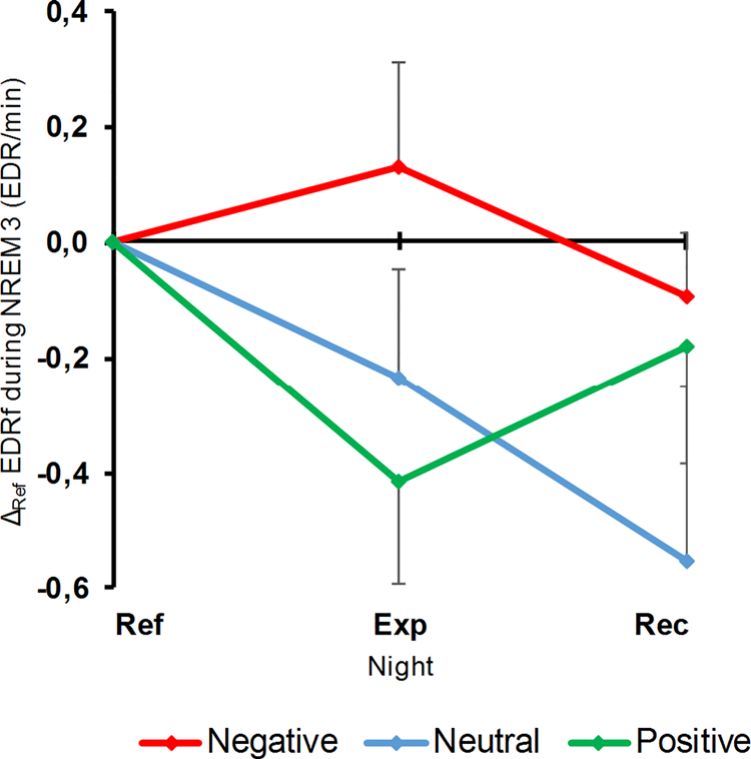
EDRf variations during NREM3, according to nights and movies. Points represent the difference (means and SEM) between each night and their corresponding Ref night. Abbreviations for nights and movie valences are the same as those of Fig 2.

During NREM3 sleep, emotional induction resulted in a valence effect on the frequency of EDR (LC: F_1,11_ = 4.89, p = 0.05; QC: F_1,11_ = 0.25, p = 0.63; Fig 6), corresponding to an increase following the negative induction whereas there was a decrease after the positive induction. This effect did not persist until the Rec night (LC: F_1,11_ = 0.12, p = 0.73), in spite of a non-significant recovery (LC: F_1,11_ = 2.00, p = 0.19; QC: F_1,11_ = 0.74, p = 0.41).

The analysis was not pursued by distinguishing the two halves of the night because the EDRf could not be calculated for some nights (12 nights with less than 2% NREM3 in the second half, 4 nights without NREM3 sleep).

During REM sleep, no effect of emotional induction on EDRf was found (LC: F_1,11_ = 0.01, p = 0.91; QC: F_1,11_ = 0.45, p = 0.51).

## Discussion

The aim of the present study was to assess whether pre-sleep emotional states affect the parameters of NREM3 and REM sleep and nocturnal sympathetic activity. Particular attention was paid to the control of the emotional induction procedure. Laboratory-made movies, that proved to be efficient at inducing congruent emotional states, were delivered just prior to an undisturbed recorded sleep period. Firstly, we found that both negative and positive emotional movies led to an increase in REM sleep. With a positive induction, the increase held during the whole night, while for a negative one, it was delayed and could be found only during the second half of the sleep period. Secondly, the valence of the pre-sleep movie also impacted the sympathetic activity during NREM3 sleep; for positive induction, a decrease in the sympathetic discharge was obtained contrasting with an increase for negative induction.

### Effect of pre-sleep emotional states on sleep architecture

The present data support a modulation of sleep architecture according to the diurnal emotional experience. The interest of these data is reinforced by the fact that the reference nights showed a classic distribution of stages during the nocturnal sleep period [52, 53]. Moreover, in contrast with previous studies examining the influence of diurnal emotions on sleep course, the present study used standardized stimulations covering the three emotional valence expressions. The validity of this procedure was confirmed by data showing that each emotional stimulation induced an emotional state of similar valence before bedtime. Despite a previous work reporting an increase in NREM3 sleep following negative pre-sleep emotional states [27], no variation in this parameter was found in the present study. As mentioned by Talamini *et al*. [27], such differential results could be explained by the high level of emotional distress of their stimulations, requiring an increase in compensatory restoring responses during the NREM3.

From a more theoretical viewpoint, the reported results are coherent with the idea of a central role of REM sleep in cross-night adaptation to emotional information (see Gruber and Cassoff [54] for a review). It is worth noting that, despite a growing interest in this topic, the impact of pre-sleep negative emotions on REM sleep remains unclear, due to contradictory data, and that the impact of positive pre-sleep emotions has been largely ignored [16] The clear increase in the REM sleep rate observed here could be related to the need to process emotional saliency, whether negative or positive, as an important dimension of stimuli underlying most behavioral adaptations [55]. Nevertheless, this increase contradicts some previous works using negative emotional movies that report perturbations (*i*.*e*. waking-up) [26], altered patterning [27], or no observed changes [25, 29, 56] in REM sleep. Some methodological differences between those studies and the current research could contribute to explaining the reported differences, such as the kind of population used (different gender ratio, cultural differences), the procedure (night to night comparison) or the movie type (a short single extract or a full movie). In fact, Baekeland *et al*. [26], Talamini *et al*. [27], Goodenough *et al*. [29] and Cluydts and Visser [25] used movies of very short duration, while Werner *et al*. [56] used full-length movies, longer than ours. If we consider the length of the movie as an analog of the exposure time to a stressor, animal studies could shed some light on this discrepancy. It is well known that in rat, stress increases REM sleep, and this effect can be influenced by different factors, notably the duration of the stressor (see Suchecki *et al*. [57] for a review). In this vein, Marinesco *et al*. [58] showed that when the stressor duration was short or long, the REM sleep increase usually obtained with a medium-length exposure could be reduced. In so far as the parallel between the stressor duration in animal and the movie duration in human is acceptable, this notion of an optimum stimulus duration to provoke an increase in REM sleep could partially explain the difference between previous reports [25–27, 29, 56] and the present data.

In addition, our data contrast with those studies emphasizing the impact of pre-sleep stress on REM sleep in human and showing a decrease in its duration or rate during the subsequent night [18, 20]. These differences must be interpreted with regard to the fact that the procedures differed in several ways. Germain *et al*. [18] and Vandekerckhove *et al*. [20] induced a negative pre-sleep state by frustrating the participant and perturbing his self-esteem, which greatly differs from the type of emotional induction applied in the present study. In this context, and again on the basis of animal studies, an explanation could be that some part of the variations in REM sleep rate is linked to the possibility of controlling the stimulus. Sanford *et al*. [59] showed that when mice could not avoid a stressor, a decrease in REM sleep occurred. However, when there was a possibility of avoiding the stressor, the reverse effect was found. In both the previous studies [18, 20], as the participant had no way of changing it, the situation could mimic an uncontrollable stressor. Consistent with animal studies, these authors obtained a decrease in the REM sleep rate. By contrast, the passive exposure to an emotional movie, as carried out in our study, can hardly be assimilated to an uncontrollable stressful situation.

Another important point is that the increase in REM sleep started earlier when the participant experienced a positive pre-sleep emotional state rather than a negative one. The REM sleep rate increased in both halves of the night following the positive movie, but only in the second half after the negative movie. This suggests that an inhibitory mechanism comes into play. In an adaptive perspective, it could be speculated that such a mechanism delays the increase in REM sleep, a particularly deep stage in which body reactivity is inhibited and the wake-up threshold is higher than in other sleep stages [60]. In a negative context, such a mechanism would preserve temporarily the possibility of reacting to an aversive stimulus. Later, *i*.*e*. in the last part of the night, it would be attenuated in order to allow REM sleep increase, required for the processing of emotional information [61, 62]. Considering the same framework, following a positive emotional experience, no inhibition takes place as no negative event is expected, and the REM sleep increase can start earlier in the night. The observed increase in REM sleep following positive stimulations in the present study is clearly in favor of our proposal.

### Effect of pre-sleep emotional states on sympathetic activity during sleep

The present data confirm the previously described EDA variations as a function of sleep stage [33–35]: EDRf was maximal during NREM3 sleep, became lower during NREM2 sleep, and attained its lowest values during REM sleep. Moreover, an increase in sympathetic activity during NREM3 sleep occurred after the negative movie, and a decrease after the positive one. This result supports the hypothesis that electrodermal discharges during sleep are influenced by pre-sleep emotional states [34, 41]. McDonald *et al*. [41] proposed that, under a pre-sleep stressful condition, the increase in electrodermal activity during sleep can be a byproduct of a sleep inhibitory mechanism, which fits the notion that higher vigilance corresponds to higher sympathetic activity. In fact, from an adaptive point of view, a slighter sleep during a period of environmental threat has an important survival value. Thus, the increased frequency in EDR we observed following negative emotional induction could reflect a mechanism limiting the fall in vigilance during sleep. In the same vein, a positive pre-sleep emotional context could indicate a safe sleep environment, attested by a decrease in sympathetic activity and an increase in REM sleep in the first half of the night. The fact that such EDA variations took place during NREM3 and not during REM sleep could be related to the idea that the inhibitory somatic mechanism inherent in REM sleep is extended to the autonomic system during this sleep stage [63]. Thus, the mediation of central variations in activation through the sympathetic channel would be restricted to NREM sleep, as observed in the current study.

Given that, in adults, NREM3 sleep mainly occurs during the first part of the night and REM sleep in the second part [64], a common mechanism could underlie the higher sympathetic activity during the NREM3 stage and the delayed increase in REM sleep, both observed after negative induction. As suggested by several studies [6, 18] associating stress stimulations, increases in norepinephrine and EDA or REM sleep variations, the noradrenergic system could be part of such a mechanism. In fact, in animal and in human, a high level of norepinephrine can inhibit the appearance of REM sleep [65–68] and increase electrodermal activity [69, 70]. Thus, following a negative emotional experience, the central noradrenergic system could be activated in order to maintain vigilance, as shown by EDA, and to shorten the episodes of inhibited reactivity, as suggested by the decrease in the REM sleep rate. The time needed for the noradrenergic system to recover its baseline would explain the observed difference between the two halves of the night to process negative information: initially, a central activation mediated by autonomic efferences and inhibiting mechanisms of REM sleep expression, followed by a reduction in such mechanisms, thus allowing the full expression of REM sleep.

### Methodological considerations

In spite of quite clear effects of pre-sleep emotional induction on sleep and sympathetic activity during the nocturnal rest period, some methodological points related to limitations and strengths of the study should be considered. Concerning limitations, though this study includes 120 nights of sleep recording, the number of participants remains low, which limits the statistical power of the data. Moreover, we focused on the sleep of young men to avoid physiological and behavioral variations in the menstrual cycle that may modulate central activation and emotional reactivity [71]. Thus, the present data cannot be extended to women or an older population. Though the diurnal activities of the participants were not fully controlled, particular attention was paid to this point, mainly through the analysis of the actiwatch and questionnaire measures, which did not reveal major specific or individual variations.

In contrast, and compared to most studies devoted to emotion-sleep interactions, the present research involved some advantageous methodological aspects. Firstly, unlike many other studies in the field of emotion, none of our participants was following a psychological cursus, which reinforces the value of the data obtained in so far as participants remained blind to the aims of the study. Secondly, in order to induce a particular emotional state, for the first time to our knowledge, three different movies, corresponding to negative, positive and neutral valence, were presented and validated as pre-sleep emotional stimulations. Though frustration or induced stress [18, 20] offers the advantage of being more ecological or realistic in some respects, a movie-based procedure strongly limits experimenter-participant interaction and, hence, appears more reliable [23, 24]. In this study, we also obtained robust results confirming that each valence induced the emotional congruent effect before the sleep period. Thirdly, the systematic recording of Ref nights enabled us to explore the potential persistence or recovery of induced emotional effects beyond Exp nights. In spite of the fact that the present results did not reveal any persistence, this methodological point could open interesting perspectives related to pathology, *i*.*e*. the persistence of highly arousing emotional experiences in the establishment of post-traumatic stress disorder [72, 73]. Finally, in the same vein as two previous studies [18, 27], the present data analysis supported the idea that sleep is a dynamic process with differential implications for emotional processing in the first and second halves of the sleep period. Accordingly, these results confirm this orientation and could help to highlight the processes of emotion-sleep relationships in future studies.

### Conclusion

The data reported here support the role of diurnal emotional experiences in the REM sleep course and, for the first time with this type of induction, highlight the impact of a pre-sleep positive emotional state. The results also confirm that nocturnal sympathetic activity is modulated by pre-sleep emotional states. To explain these effects, we hypothesized two mechanisms. The first, related to emotional arousal, would increase REM sleep, likely to facilitate the processing of emotional experience and prepare the individual for potential emotional challenges taking place in the future. The second, related to emotional valence, would be temporarily activated in the case of a negative emotional experience, together with an activation of the sympathetic system, and would lead to a delayed REM sleep. Its function would be to adjust, during the first part of the night, the level of vigilance to potential threats from the environment, *i*.*e*. to preserve body reactivity and optimize adaptation. We can also suppose that the reported REM sleep changes could aim to modulate the post-sleep emotional reactivity during the following day. Thus, the impact of pre-sleep emotional states on post-sleep emotional reactivity could be a heuristic challenge in future studies.

Finally, according to our results, a negative pre-sleep emotional state could lead to an increase in sympathetic activity during the following sleep period and this increase is related to changes in the sleep course. These observations could help to understand sleep disturbances, such as secondary comorbid insomnia, which can occur notably in mental disorders, like anxiety [74, 75]. The disturbances in these pathologies are thought to be linked to a difficulty in disengaging emotional and body arousal, which disrupts the initiation and course of sleep (see Baglioni *et al*. [76] for a review). Accordingly, Kahn *et al*. [77] suggested that there could be an interplay between concomitant emotional arousal and sleep problems, which maintain each other, creating a “vicious circle” [62, 77]. In a complementary way, we also reported a decrease in sympathetic activity, induced by pre-sleep positive emotional states, during the early part of the night; this reveals the decrease in the arousal level and thus the possibility of breaking the hypothesized circle. In this way, modulating the arousal during the early sleep course by using pre-sleep positive stimulations could lead to adjusting sleep patterns impacted by diurnal emotions. Although only healthy participants were involved here, the present study opens new avenues for potential practices designed to alleviate sleep disturbances.

## References

[1] Ong JC, Carde NB, Gross JJ, Manber R. A two-dimensional approach to assessing affective states in good and poor sleepers. Journal of sleep research. 2011;20(4):606–10. 10.1111/j.1365-2869.2011.00907.x 21244540 PMC3119723

[2] Galambos NL, Howard AL, Maggs JL. Rise and Fall of Sleep Quantity and Quality With Student Experiences Across the First Year of University. Journal of Research on Adolescence. 2011;21(2):342–9.

[3] Settineri S, Vitetta A, Mento C, Fanara G, Silvestri R, Tati F, et al Construction of a telephone interview to assess the relationship between mood and sleep in adolescence. Neurological sciences: official journal of the Italian Neurological Society and of the Italian Society of Clinical Neurophysiology. 2010;31(4):459–65. 10.1007/s10072-010-0255-z 20414705

[4] Grano N, Vahtera J, Virtanen M, Keltikangas-Jarvinen L, Kivimaki M. Association of hostility with sleep duration and sleep disturbances in an employee population. International journal of behavioral medicine. 2008;15(2):73–80. 10.1080/10705500801929510 18569125

[5] Fortunato VJ, Harsh J. Stress and sleep quality: The moderating role of negative affectivity. Personality and Individual Differences. 2006;41(5):825–36.

[6] Mezick EJ, Matthews KA, Hall M, Kamarck TW, Buysse DJ, Owens JF, et al Intra-individual variability in sleep duration and fragmentation: associations with stress. Psychoneuroendocrinology. 2009;34(9):1346–54. 10.1016/j.psyneuen.2009.04.005 19450933 PMC2743778

[7] Lundh LG, Broman JE. Insomnia as an interaction between sleep-interfering and sleep-interpreting processes. Journal of psychosomatic research. 2000;49(5):299–310. 11164054 10.1016/s0022-3999(00)00150-1

[8] Hicks RA, Garcia ER. Level of stress and sleep duration. Perceptual and motor skills. 1987;64(1):44–6. 3562191 10.2466/pms.1987.64.1.44

[9] Akerstedt T, Kecklund G, Axelsson J. Impaired sleep after bedtime stress and worries. Biological psychology. 2007;76(3):170–3. 17884278 10.1016/j.biopsycho.2007.07.010

[10] Cartwright RD, Wood E. Adjustment disorders of sleep: the sleep effects of a major stressful event and its resolution. Psychiatry research. 1991;39(3):199–209. 1798820 10.1016/0165-1781(91)90088-7

[11] Brand S, Beck J, Hatzinger M, Harbaugh A, Ruch W, Holsboer-Trachsler E. Associations between satisfaction with life, burnout-related emotional and physical exhaustion, and sleep complaints. The world journal of biological psychiatry: the official journal of the World Federation of Societies of Biological Psychiatry. 2010;11(5):744–54. 10.3109/15622971003624205 20331383

[12] Steptoe A, O'Donnell K, Marmot M, Wardle J. Positive affect, psychological well-being, and good sleep. Journal of psychosomatic research. 2008;64(4):409–15. 10.1016/j.jpsychores.2007.11.008 18374740

[13] Troxel WM, Buysse DJ, Hall M, Matthews KA. Marital happiness and sleep disturbances in a multi-ethnic sample of middle-aged women. Behavioral sleep medicine. 2009;7(1):2–19. 10.1080/15402000802577736 19116797 PMC2654623

[14] Ryff CD, Singer BH, Dienberg Love G. Positive health: connecting well-being with biology. Philosophical transactions of the Royal Society of London Series B, Biological sciences. 2004;359(1449):1383–94. 15347530 10.1098/rstb.2004.1521 PMC1693417

[15] Kim E-J, Dimsdale JE. The effect of psychosocial stress on sleep: a review of polysomnographic evidence. Behavioral sleep medicine. 2007;5(4):256–78. 17937582 10.1080/15402000701557383 PMC4266573

[16] Deliens G, Gilson M, Peigneux P. Sleep and the processing of emotions. Experimental brain research Experimentelle Hirnforschung Experimentation cerebrale. 2014;232(5):1403–14. 10.1007/s00221-014-3832-1 24449011

[17] Kobayashi T, Ishikawa T, Arakawa K. Effects of daytime activity upon the timing of REM sleep periods during a night. Psychiatry and clinical neurosciences. 1998;52(2):130–1. 9628110 10.1111/j.1440-1819.1998.tb00989.x

[18] Germain A, Buysse DJ, Ombao H, Kupfer DJ, Hall M. Psychophysiological reactivity and coping styles influence the effects of acute stress exposure on rapid eye movement sleep. Psychosomatic medicine. 2003;65(5):857–64. 14508032 10.1097/01.psy.0000079376.87711.b0

[19] Kecklund G, Akerstedt T. Apprehension of the subsequent working day is associated with a low amount of slow wave sleep. Biological psychology. 2004;66(2):169–76. 15041138 10.1016/j.biopsycho.2003.10.004

[20] Vandekerckhove M, Weiss R, Schotte C, Exadaktylos V, Haex B, Verbraecken J, et al The role of presleep negative emotion in sleep physiology. Psychophysiology. 2011;48(12):1738–44. 10.1111/j.1469-8986.2011.01281.x 21895689

[21] Cohen DB. Eye movements during REM sleep: the influence of personality and presleep conditions. Journal of personality and social psychology. 1975;32(6):1090–3. 175146

[22] Vein AM, Sudakov KV, Levin YI, Yumatov EA, Strygin KN, Kovrov GV. Stages of sleep after psychoemotional tension: the individual character of changes. Neuroscience and behavioral physiology. 2002;32(5):513–8. 12403003 10.1023/a:1019859606601

[23] Rosenthal R. Experimenter effects in behavioral research. New York: Appleton-Century-Crofts; 1966.

[24] Gilet A-L. [Mood induction procedures: a critical review]. L'Encephale. 2008;34(3):233–9. 10.1016/j.encep.2006.08.003 18558143

[25] Cluydts R, Visser P. Mood and sleep. II. Effects of aversive pre-sleep stimulation. Waking and sleeping. 1980;4(3):199–203. 7281631

[26] Baekeland F, Koulack D, Lasky R. Effects of a stressful presleep experience on electroencephalograph-recorded sleep. Psychophysiology. 1968;4(4):436–43. 5662813 10.1111/j.1469-8986.1968.tb02784.x

[27] Talamini LM, Bringmann LF, de Boer M, Hofman WF. Sleeping worries away or worrying away sleep? Physiological evidence on sleep-emotion interactions. PloS one. 2013;8(5):e62480 10.1371/journal.pone.0062480 23671601 PMC3641038

[28] Lauer C, Riemann D, Lund R, Berger M. Shortened REM latency: a consequence of psychological strain? Psychophysiology. 1987;24(3):263–71. 3602281 10.1111/j.1469-8986.1987.tb00293.x

[29] Goodenough DR, Witkin HA, Koulack D, Cohen H. The effects of stress films on dream affect and on respiration and eye-movement activity during Rapid-Eye-Movement sleep. Psychophysiology. 1975;12(3):313–20. 168607 10.1111/j.1469-8986.1975.tb01298.x

[30] Kimata H. Viewing humorous film improves nighttime wakening in children with atopic dermatitis. Indian pediatrics. 2007;44(4):281–5. 17468523

[31] Kreibig SD. Autonomic nervous system activity in emotion: a review. Biological psychology. 2010;84(3):394–421. 10.1016/j.biopsycho.2010.03.010 20371374

[32] Freixa i Baqué E, Chevalier B, Grubar JC, Lambert C, Lancry A, Leconte P, et al Spontaneous electrodermal activity during sleep in man: an intranight study. Sleep. 1983;6(1):77–81. 6844801 10.1093/sleep/6.1.77

[33] Freixa i Baqué E. Reliability of spontaneous electrodermal activity in humans as a function of sleep stages. Biological psychology. 1983;17(2–3):137–43. 6640011 10.1016/0301-0511(83)90014-5

[34] Lester BK, Burch NR, Dossett RC. Nocturnal EEG-GSR profiles: the influence of presleep states. Psychophysiology. 1967;3(3):238–48. 6038667 10.1111/j.1469-8986.1967.tb02701.x

[35] Johnson LC, Lubin A. Spontaneous electrodermal activity during waking and sleeping. Psychophysiology. 1966;3(1):8–17. 5942878 10.1111/j.1469-8986.1966.tb02673.x

[36] Freixa i Baqué E, Delerm B, Roy JC. Reliability of spontaneous electrodermal activity in the cat as a function of waking and sleep stages. Biological psychology. 1980;10(3):219–24. 7470520 10.1016/0301-0511(80)90017-4

[37] Delerm B, Delsaut M, Freixa i Baqué E. Stability of spontaneous electrodermal activity in the kitten. Biological psychology. 1981;12(4):299–304. 7340894 10.1016/0301-0511(81)90003-x

[38] Critchley HD. Electrodermal responses: what happens in the brain. The Neuroscientist: a review journal bringing neurobiology, neurology and psychiatry. 2002;8(2):132–42. 10.1177/107385840200800209 11954558

[39] Sequeira H, Hot P, Silvert L, Delplanque S. Electrical autonomic correlates of emotion. International journal of psychophysiology: official journal of the International Organization of Psychophysiology. 2009;71(1):50–6. 18723054 10.1016/j.ijpsycho.2008.07.009

[40] Sano A, Picard RW, editors. Recognition of sleep dependent memory consolidation with multi-modal sensor data. Body Sensor Networks (BSN), 2013 IEEE International Conference on; 2013 6–9 May 2013.

[41] McDonald DG, Shallenberger HD, Koresko RL, Kinzy BG. Studies of spontaneous electrodermal responses in sleep. Psychophysiology. 1976;13(2):128–34. 1257374 10.1111/j.1469-8986.1976.tb00087.x

[42] Weise S, Ong J, Tesler NA, Kim S, Roth WT. Worried sleep: 24-h monitoring in high and low worriers. Biological psychology. 2013;94(1):61–70. 10.1016/j.biopsycho.2013.04.009 23643927

[43] Kushniruk A, Rustenburg J, Ogilvie R. Psychological correlates of electrodermal activity during REM sleep. Sleep. 1985;8(2):146–54. 4012157 10.1093/sleep/8.2.146

[44] Schaefer A, Nils F, Sanchez X, Philippot P. Assessing the effectiveness of a large database of emotion-eliciting films: A new tool for emotion researchers. Cognition & emotion. 2010;24(7):1153–72.

[45] Lang PJ. The varieties of emotional experience: a meditation on James-Lange theory. Psychological review. 1994;101(2):211–21. 8022956 10.1037/0033-295x.101.2.211

[46] Kawahito J, Otsuka Y, Kaida K, Nakata A. [Reliability and validity of the Japanese version of 20-item Positive and Negative Affect Schedule]. Hiroshima Psychological Research. 2012;11:225–40.

[47] Bradley MM, Lang PJ. Measuring emotion: the Self-Assessment Manikin and the Semantic Differential. Journal of behavior therapy and experimental psychiatry. 1994;25(1):49–59. 7962581 10.1016/0005-7916(94)90063-9

[48] Jasper H. Report of the committee on methods of clinical examination in electroencephalography: 1957. Electroencephalography and clinical neurophysiology. 1958;10(2):370–5.

[49] Agnew HW, Webb WB, Williams RL. The first night effect: an EEG study of sleep. Psychophysiology. 1966;2(3):263–6. 5903579 10.1111/j.1469-8986.1966.tb02650.x

[50] Lorenzo J-L, Barbanoj M-J. Variability of sleep parameters across multiple laboratory sessions in healthy young subjects: the "very first night effect". Psychophysiology. 2002;39(4):409–13. 12212632 10.1017/S0048577202394010

[51] Iber C, Ancoli-Israel S, Chesson AL, Quan SF. The AASM—Manual for the Scoring of Sleep and Associted Events: Rules, terminology and technical specifications. American Academy of Sleep Medicine. 2007.

[52] Ohayon MM, Carskadon MA, Guilleminault C, Vitiello MV. Meta-analysis of quantitative sleep parameters from childhood to old age in healthy individuals: developing normative sleep values across the human lifespan. Sleep. 2004;27(7):1255–73. 15586779 10.1093/sleep/27.7.1255

[53] Carskadon MA, Dement WC. Chapter 2—Normal Human Sleep: An Overview In: Dement MHKRC, editor. Principles and Practice of Sleep Medicine (Fifth Edition). Philadelphia: W.B. Saunders; 2011 p. 16–26.

[54] Gruber R, Cassoff J. The interplay between sleep and emotion regulation: conceptual framework empirical evidence and future directions. Current psychiatry reports. 2014;16(11):500 10.1007/s11920-014-0500-x 25200984

[55] Walker MP. The role of sleep in cognition and emotion. Annals of the New York Academy of Sciences. 2009;1156:168–97. 10.1111/j.1749-6632.2009.04416.x 19338508

[56] Werner GG, Schabus M, Blechert J, Kolodyazhniy V, Wilhelm FH. Pre- to postsleep change in psychophysiological reactivity to emotional films: Late-night REM sleep is associated with attenuated emotional processing. Psychophysiology. 2015;52(6):813–25. 10.1111/psyp.12404 25588962

[57] Suchecki D, Tiba PA, Machado RB. REM Sleep Rebound as an Adaptive Response to Stressful Situations. Frontiers in neurology. 2012;3:41 10.3389/fneur.2012.00041 22485105 PMC3317042

[58] Marinesco S, Bonnet C, Cespuglio R. Influence of stress duration on the sleep rebound induced by immobilization in the rat: a possible role for corticosterone. Neuroscience. 1999;92(3):921–33. 10426533 10.1016/s0306-4522(99)00045-7

[59] Sanford LD, Yang L, Wellman LL, Liu X, Tang X. Differential effects of controllable and uncontrollable footshock stress on sleep in mice. Sleep. 2010;33(5):621–30. 20469804 10.1093/sleep/33.5.621 PMC2864877

[60] Ermis U, Krakow K, Voss U. Arousal thresholds during human tonic and phasic REM sleep. Journal of sleep research. 2010;19(3):400–6. 10.1111/j.1365-2869.2010.00831.x 20477954

[61] Walker MP. Sleep, memory and emotion. Progress in brain research. 2010;185:49–68. 10.1016/B978-0-444-53702-7.00004-X 21075233

[62] Walker MP, van der Helm E. Overnight therapy? The role of sleep in emotional brain processing. Psychological bulletin. 2009;135(5):731–48. 10.1037/a0016570 19702380 PMC2890316

[63] Antrobus JS, Wamsley EJ. Sleep mentation in REM and NREM: A neurocognitive perspective. In: Ed. Squire AP L.R., editor. Enclyclopedia of Neuroscience. Vol. 8 Amsterdam2009. p. pp 1021–6.

[64] Bes F, Schulz H, Navelet Y, Salzarulo P. The distribution of slow-wave sleep across the night: a comparison for infants, children, and adults. Sleep. 1991;14(1):5–12. 1811320 10.1093/sleep/14.1.5

[65] Dunleavy DL, Brezinova V, Oswald I, Maclean AW, Tinker M. Changes during weeks in effects of tricyclic drugs on the human sleeping brain. The British journal of psychiatry: the journal of mental science. 1972;120(559):663–72. 4339630 10.1192/bjp.120.559.663

[66] Ross RJ, Gresch PJ, Ball WA, Sanford LD, Morrison AR. REM sleep inhibition by desipramine: evidence for an alpha-1 adrenergic mechanism. Brain research. 1995;701(1–2):129–34. 8925274 10.1016/0006-8993(95)00984-x

[67] Mallick BN, Singh A, Khanday MA. Activation of inactivation process initiates rapid eye movement sleep. Progress in neurobiology. 2012;97(3):259–76. 10.1016/j.pneurobio.2012.04.001 22521402

[68] Kraft NO, Inoue N, Mizuno K, Ohshima H, Murai T, Sekiguchi C. Physiological changes, sleep, and morning mood in an isolated environment. Aviation, space, and environmental medicine. 2002;73(11):1089–93. 12433232

[69] Yamamoto K, Ozawa N, Shinba T, Hoshino T. Functional influence of the central noradrenergic system on the skin conductance activity in rats. Schizophrenia research. 1994;13(2):145–50. 7986771 10.1016/0920-9964(94)90095-7

[70] Süer C, Ozesmi C, Temoçin S, Doğan P, Ciliv G. The effects of immobilization stress on electrodermal activity and brain catecholamine levels in rats. The International journal of neuroscience. 1992;65(1–4):91–101. 1341695 10.3109/00207459209003281

[71] Fleischman DS, Navarrete CD, Fessler DMT. Oral contraceptives suppress ovarian hormone production. Psychological science. 2010;21(5):750–2; author reply 3. 10.1177/0956797610368062 20483856

[72] Auxéméry Y. [Posttraumatic stress disorder (PTSD) as a consequence of the interaction between an individual genetic susceptibility, a traumatogenic event and a social context]. L'Encephale. 2012;38(5):373–80. 10.1016/j.encep.2011.12.003 23062450

[73] Philbert J, Pichat P, Beeské S, Decobert M, Belzung C, Griebel G. Acute inescapable stress exposure induces long-term sleep disturbances and avoidance behavior: a mouse model of post-traumatic stress disorder (PTSD). Behavioural brain research. 2011;221(1):149–54. 10.1016/j.bbr.2011.02.039 21377492

[74] Tsypes A, Aldao A, Mennin DS. Emotion dysregulation and sleep difficulties in generalized anxiety disorder. Journal of anxiety disorders. 2013;27(2):197–203. 10.1016/j.janxdis.2013.01.008 23474909

[75] Pigeon WR, Gallegos AM. Posttraumatic Stress Disorder and Sleep. Sleep medicine clinics. 2015;10(1):41–8. 10.1016/j.jsmc.2014.11.010 26055672

[76] Baglioni C, Spiegelhalder K, Lombardo C, Riemann D. Sleep and emotions: a focus on insomnia. Sleep medicine reviews. 2010;14(4):227–38. 10.1016/j.smrv.2009.10.007 20137989

[77] Kahn M, Sheppes G, Sadeh A. Sleep and emotions: Bidirectional links and underlying mechanisms. International journal of psychophysiology: official journal of the International Organization of Psychophysiology. 2013. 10.1016/j.ijpsycho.2013.05.010 23711996

